# Synthesis of Polyether Carboxylate and the Effect of Different Electrical Properties on Its Viscosity Reduction and Emulsification of Heavy Oil

**DOI:** 10.3390/polym15143139

**Published:** 2023-07-24

**Authors:** Junqi Wang, Ruiqing Liu, Bo Wang, Zhigang Cheng, Chengkun Liu, Yiwen Tang, Junfeng Zhu

**Affiliations:** 1 The Key Laboratory of Well Stability and Fluid & Rock Mechanics in Oil and Gas Reservoir of Shaanxi Province, Xi’an Shiyou University, Xi’an 710065, China; wjqi@xsyu.edu.cn; 2 Shaanxi Key Research Laboratory of Chemical Additives, College of Chemistry and Chemical Engineering, Shaanxi University of Science and Technology, Xi’an 710021, China; 201909020720@sust.edu.cn (R.L.); 201909020708@sust.edu.cn (Y.T.); 3 The Fourth Oil Production Factory of PetroChina Changqing Oilfield Company, Jingbian 718500, China; wangbo9_cq@petrochina.com.cn; 4 The Third Gas Production Plant of PetroChina Changqing Oilfield Company, Xi’an 710021, China; chengg1_cq@petrochina.com.cn; 5 The First Gas Production Plant of PetroChina Changqing Oilfield Company, Xi’an 710021, China; lkfeng_cq@petrochina.com.cn

**Keywords:** different electrical properties, polyether carboxylate viscosity reducers, surfactants, viscosity reduction, heavy oil

## Abstract

Heavy oil exploitation needs efficient viscosity reducers to reduce viscosity, and polyether carboxylate viscosity reducers have a significant viscosity reduction effect on heavy oil. Previous work has studied the effect of different side chain lengths on this viscosity reducer, and now a series of polyether carboxylate viscosity reducers, including APAD, APASD, APAS, APA, and AP5AD (the name of the viscosity reducer is determined by the name of the desired monomer), with different electrical properties have been synthesized to investigate the effect of their different electrical properties on viscosity reduction performance. Through the performance tests of surface tension, contact angle, emulsification, viscosity reduction, and foaming, it was found that APAD viscosity reducers had the best viscosity reduction performance, reducing the viscosity of heavy oil to 81 mPa·s with a viscosity reduction rate of 98.34%, and the worst viscosity reduction rate of other viscosity reducers also reached 97%. Additionally, APAD viscosity reducers have the highest emulsification rate, and the emulsion formed with heavy oil is also the most stable. The net charge of APAD was calculated from the molar ratio of the monomers and the total mass to minimize the net charge. While the net charge of other surfactants was higher. It shows that the amount of the surfactant’s net charge affects the surfactant’s viscosity reduction effect, and the smaller the net charge of the surfactant itself, the better the viscosity reduction effect.

## 1. Introduction

Petroleum oil plays a significant role in modern industrial production; in the meantime, with the development of the economy and technology, oil consumption is increasing at a constant rate [1]. In order to meet the consumption of oil, it is necessary to extract oil continuously, especially when oil extraction has entered the third stage [2]. Heavy oil, as one of the objects of tertiary oil extraction, has the characteristics of high viscosity and poor fluidity [3], which leads to difficulties in heavy oil extraction [4]. Heavy oil contains a large number of asphaltenes and gums, and the complex aggregation structure formed by the mutual accumulation and adsorption of these two, together with the action of hydrogen bonds and the presence of van der Waals forces, is what leads to the large viscosity of heavy oil [3,5,6]. However, chemically enhanced oil recovery (cEOR) can be strengthened by the use of chemical additives [7,8,9,10]. In cEOR, common chemical additives are surfactants, polymers, bases, and their complex systems. Surfactants are used to form stable emulsions to improve oil drive efficiency by reducing the interfacial tension between oil and water and changing the wettability of the rock [11,12,13,14,15]. Polymers are used to improve oil-repelling efficiency by increasing the viscosity of the repellent phase to reduce the viscous plugging ratio [10,16]. Alkalis are used to modify the mobility of heavy oil by decreasing the interfacial tension between oil and water [17,18]. In addition, some scholars have discovered new chemical additives that can also improve oil-repelling efficiency, such as the use of nanofluids for oil repelling [19,20] and the application of viscoelastic surfactants [21].

Among the general chemically enhanced oil drive methods, alkali drive is used infrequently and is not suitable for reservoirs with high mineralization [22]. Meanwhile, the complex system is unstable and prone to chromatographic separation and chemical reagent loss during flow [23], and the general surfactants contain anionic surfactants, cationic surfactants, amphoteric surfactants, and nonionic surfactants [24], in a wide variety, each with its own advantages and disadvantages. Therefore, this paper studies polymers with surfactant properties, i.e., polymeric surfactants, which are stable and possess the same surface properties as general surfactants.

Some researchers have worked on polymeric surfactants as viscosity reducers for heavy oil. Li Juan et al. prepared a high molecular weight polymeric surfactant, an anionic long-branched polymeric viscosity reducer (AAGAS) containing glycidyl methacrylate (GMA), and the study showed that this anionic polymeric surfactant has good viscosity reduction ability and surface interfacial activity with an effect that is superior to that of commercial small molecule surfactants [25]. Agam Duma Kalista Wibowo et al. used palm oil methyl ester to synthesize a polymeric surfactant and used it for enhanced oil recovery. The results showed that although it could not significantly reduce the interfacial tension, it could still improve the oil drive efficiency [26]. Jana et al. investigated the viscosity reduction effect of polyoxyethylene sorbitol monooleate on heavy oil, and the study showed that the effect was both significant and more than 95% under optimal conditions [27]. Kang Xin et al. used amphiphilic polymers and surfactants to form a heavy oil cold recovery self-emulsifying complex system, and the study showed that the system effectively increased the stability of the emulsion [28]. Chen Hong and Zhang Lan explored the emulsification performance of sulfonate-type polymeric surfactants and reached the conclusion that the emulsification performance increased with an increase in polymeric surfactant concentration [29]. These studies show that polymeric surfactants are promising for the viscosity reduction and emulsification of heavy oil. Whereas the effect of the polymeric surfactant structure and electrical properties on its performance still needs further research.

In our previous work [30], we synthesized polyether carboxylate viscosity reducers with different side chain lengths, performed viscosity reduction and emulsification performance tests, and achieved the optimal structures and reaction conditions. However, up to now, the effect on electrical properties of polyether carboxylate has been scarcely studied systematically, although electrical properties have a potential impact on viscosity reduction in heavy oil [31]. In this work, a series of polyether carboxylate viscosity reducers with different electrical properties have been synthesized to investigate the effect of their different electrical properties on viscosity reduction performance. We changed the electrical properties of the polyether carboxylate based on the optimal structures to enhance the performance of viscosity reducers.

## 2. Materials and Methods

### 2.1. Materials

Allyl polyoxyethylene ether 600 (APEG600) (Tianjin Damao Chemical Reagent Factory, Tianjin, China); acrylic acid (AA) (Tianjin Damao Chemical Reagent Factory); sodium *p*-styrene sulfonate (SSS) (Aladdin, Hong Kong, China); dimethyldiallyl ammonium chloride (DMDACC); sodium bisulfite (Tianjin Damao Chemical Reagent Factory); potassium persulfate (Tianjin Damao Chemical Reagent Factory); sodium hydroxide (Tianjin Damao Chemical Reagent Factory); potassium bromide (Tianjin Damao Chemical Reagent Factory); deuterated water (Shanghai Maclean Biochemical, Shanghai, China); and ethanol (Tianjin Damao Chemical Reagent Factory) were used in the study.

### 2.2. Synthesis

#### 2.2.1. Synthesis of APAS

Acrylic acid, sodium *p*-styrenesulfonate, and allyl polyoxyethylene ether were weighed according to the molar ratio of 4:1:1. The total mass of the three monomers was 30 g. Sodium bisulfite and potassium persulfate were weighed according to 1 wt% and 3 wt% of the total weight of the monomers, respectively. The above solids were dissolved into solutions using deionized water. Allyl polyoxyethylene ether, sodium bisulfite solution, and deionized water were put in a three-necked flask. Meanwhile, the flask was heated in an oil bath, stirred with a magnet, and purged of nitrogen to exhaust the air in the reaction device. When the flask temperature was raised to 80 °C, the solutions of acrylic acid, sodium *p*-styrenesulfonate, and potassium persulfate were dropped into the flask using constant-pressure dropping funnels, respectively. The dropwise addition time of acrylic acid and sodium *p*-styrenesulfonate was 2 h, and the dropwise addition time of potassium persulfate was 2.5 h. After the dropwise addition, the reaction was stirred and kept at 80 °C for another 5 h. After that, the product was cooled to room temperature, and the pH was adjusted to around 7-8 with a 20 wt% sodium hydroxide solution. The resulting solution was the polyether carboxylate viscosity reducer, APAS [30].

#### 2.2.2. Synthesis of APASD

Using diallyldimethylammonium chloride to replace sodium *p*-styrenesulfonate, diallyldimethylammonium chloride was weighed at 6% of the total mass of the monomer [32]. Other chemical reagents and operations were the same as with the synthesis of APAS. The resulting solution was the polyether carboxylate viscosity reducer, APASD.

#### 2.2.3. Synthesis of APAD

Acrylic acid, allyl polyoxyethylene ether, and dimethyldiallyl ammonium chloride were weighed at a 4:1 molar ratio, 6% of total monomer mass, and 30 g of total monomer weight. The above solids were dissolved into solutions using deionized water, and the polyether carboxylate viscosity reducer, APAD, was obtained according to the same synthesis method used with APAS.

#### 2.2.4. Synthesis of APA

Acrylic acid and allyl polyoxyethylene ether were weighted according to a molar ratio of 4:1, and the total weight of the monomer was 30 g. The above solids were dissolved into solutions using deionized water, and the polyether carboxylate viscosity reducer, APA, was obtained according to the same synthesis method used with APAS.

#### 2.2.5. Synthesis of AP5AD

Acrylic acid, allyl polyoxyethylene ether, and dimethyldiallyl ammonium chloride were weighed at a molar ratio of 5:1, 6% of the total monomer mass, and 30 g of the total monomer weight. The above solids were dissolved into solutions using deionized water, and the polyether carboxylate viscosity reducer, AP5AD, was obtained according to the same synthesis method used with APAS.

**Figure 1:**
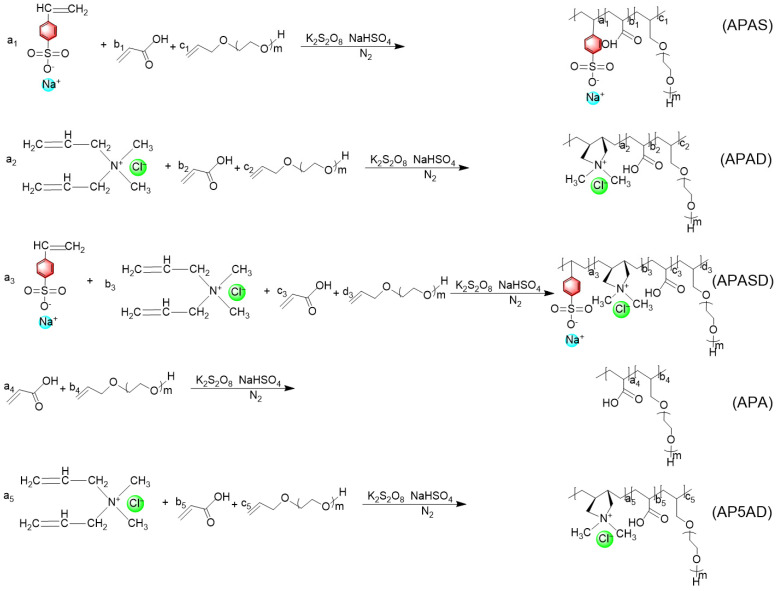
Five kinds of polyether carboxylate viscosity-reducing agent synthesis reaction formulas. In the diagram, a, b, c, and d represent the amount of each monomer.

The reaction equation is shown in Figure 1 below:

### 2.3. Characterization

Some of the synthesized samples were extracted with ethanol to remove impurities and dried before characterization.

The samples were tested by the infrared spectrometer EQUI NX55 of Brucher, Germany. Dried polyether carboxylate viscosity-reducing agent powder was mixed and ground with potassium bromide powder, dried, and processed at 100 °C. The thin slices with 90% light transmittance were prepared by the press method, and the test wave number range was from 400 to 4000 cm^−1^.

Using a Bruker Varian Inova 500 NB 600 MHz NMR hydrogen spectrometer, 5 mg of dried polyether carboxylate viscosity reducer was dissolved in 10 mL of deuterated water for characterization tests in an NMR system.

### 2.4. Determination of Heavy Oil Properties

The physicochemical properties and composition of the heavy oil were determined by GC-MS with dichloromethane as the solvent.

### 2.5. Surface Performance Testing

#### 2.5.1. Surface Tension Test

The surface tension of polyether carboxylate viscosity reducers was tested using a DCAT21 surface interfacial tension meter. Solutions of different mass concentrations were configured, and the surface tension was determined by the platinum sheet method at room temperature.

#### 2.5.2. Contact Angle Test

The contact angle of polyether carboxylate viscosity reducers was measured using a contact angle meter from Deffy East, Germany. The North China heavy oil was coated on the slide evenly, and then solutions of different mass concentrations were configured, and the contact angle of the sample solution droplets onto the surface of the heavy oil was measured at room temperature using the contact angle meter.

#### 2.5.3. Water Distribution Performance Test

It was configured with 10 mL of 0.1% polyether carboxylate viscosity reducer solution, mixed with edible oil in a ratio of 1:1 by volume, shaken with a stoppered measuring cylinder to maintain the same amplitude, shaken 100 times, and the time required to precipitate 5 mL of aqueous solution and the volume of the final precipitated aqueous solution were recorded.

#### 2.5.4. Foaming Performance Test

It was configured with 20 mL of polyether carboxylate viscosity reducer solution with different mass fractions and stirred for 10 s at 8000 r/min of emulsifier. After stirring, the foam height was recorded immediately.

### 2.6. Viscosity Reduction and Emulsification Performance Test

#### 2.6.1. Viscosity Reduction Test

The viscosity was determined by using the digital display viscometer produced by Brookfield Co. (Toronto, ON, Canada). The viscosity was determined by mixing 25 mL of 0.5% polyether carboxylate solution with North China heavy oil in a ratio of 1:1 by volume, heating in a water bath at 50 °C for 15 min, stirring with an emulsifier (D-160) at 8000 r/min for 4 min, and then standing for 1 min to determine the viscosity at that temperature. An equal volume of heavy oil was taken under the same conditions to determine its viscosity as a control group.

#### 2.6.2. Emulsification Speed Test

It was prepared with 10 mL of polyether carboxylate viscosity reducer solutions of different mass fractions, mixed with North China heavy oil at a volume ratio of 1:1, heated in a water bath at 50 °C for 15 min, then stirred with the emulsifier at 8000 r/min for 2.5 min, and the volume of the emulsion was read after standing for 1 min.

$$\mathrm{Emulsification}\;\mathrm{speed}=\frac{\mathrm{emulsion}\;\mathrm{volume}}{\mathrm{stirring}\;\mathrm{time}}$$

where the emulsion volume is in milliliters and the stirring time is in minutes.

#### 2.6.3. Emulsion Stability Testing

It was configured with 10 mL of polyether carboxylate viscosity reducer solutions of different mass fractions, mixed with North China heavy oil at a volume ratio of 1:1, heated in a water bath at 50 °C for 15 min, then stirred with an emulsifier at 8000 r/min for 2.5 min, and left for 10 min before reading the volume of the precipitated aqueous solution.

$$\mathrm{Water}\;\mathrm{separation}\;\mathrm{rate}=\frac{\;\mathrm{volume}\;\mathrm{of}\;\mathrm{precipitated}\;\mathrm{water}}{(\mathrm{emulsion}\;\mathrm{volume}+\mathrm{volume}\;\mathrm{of}\;\mathrm{precipitated}\;\mathrm{water})\;}\;$$

where the volume is in milliliters.

## 3. Results and Discussion

### 3.1. Characterization

#### 3.1.1. FT-IR Spectroscopy

**Figure 2:**
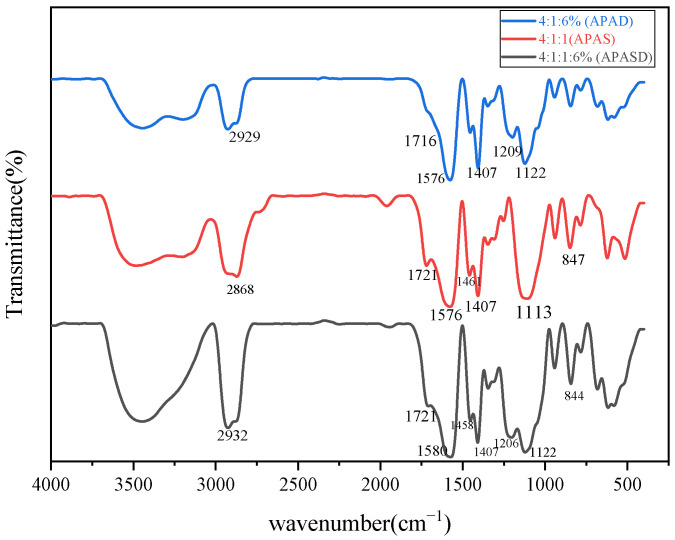
Infrared spectra of three different viscosity-reducing agents.

The infrared spectra of three surfactants, APAD, APAS, and APASD, are shown in Figure 2. From Figure 2, it can be seen that all three products have strong and broad absorption peaks in the range of 2850~2960 cm^−1^, which are the stretching vibrations of saturated C-H bonds. They all have absorption peaks near 1720 cm^−1^, which are judged to be the stretching vibrations of C=O bonds of carboxyl groups. APAS and APASD have absorption peaks near 1580, 1450, and 840 cm^−1^, which are judged to be the backbone vibration of the benzene ring and the para-disubstitution absorption peak of the benzene ring, respectively. APAD and APASD have absorption peaks near 1200 cm^−1^, which are the absorption peaks of C-N bond stretching vibration. All three have strong absorption peaks near 1100 cm^−1^, which are judged to be the absorption peaks of C-O-C. After the analysis, it can be tentatively judged that the synthesized products are the target products.

#### 3.1.2. ^1^HNMR

**Figure 3:**
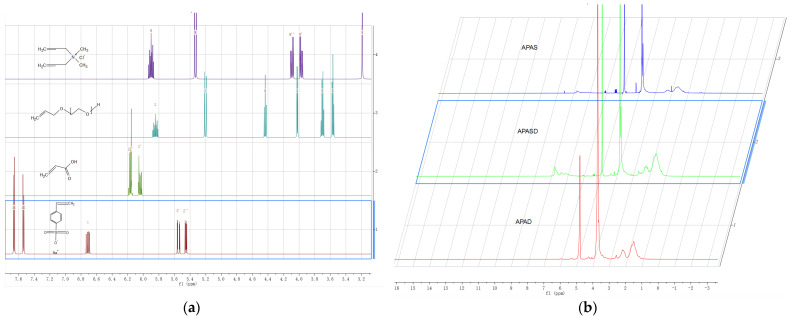
(**a**) ^1^HNMR prediction of the reaction monomer; (**b**) ^1^HNMR of the three different viscosity-reducing agents.

The NMR characterization of the three surfactants, APAS, APASD, and APAD, is shown in Figure 3 below. Comparison of the NMR prediction maps of each monomer with the NMR maps of the samples shows that the three samples have no absorption peaks at δ = 6.7 and 5.5 ppm, indicating complete reaction of the C=C bond of sodium p-styrenesulfonate; no absorption peaks near δ = 6.3 ppm, indicating complete reaction of the C=C bond of acrylic acid; no absorption peaks near δ = 5.7 and 5.3 ppm, indicating complete reaction of the C=C bond of APEG; and no absorption peak near δ = 5.9 and 5.3 ppm, indicating complete reaction of DMDACC. APAD has no absorption peak between δ = 7~8 ppm, indicating no para-disubstituted benzene ring, while APAS and APASD both have double peaks between δ = 7~8 ppm, suggesting both contain a para-disubstituted benzene ring. APAD and APASD have obvious absorption peaks between δ = 2~3 ppm, while APAS does not, suggesting that APAD and APASD have N-CH_3_ of H. All three have absorption peaks around δ = 4 ppm, which are peaks of C out of APEG near the C=C double bond; strong absorption peaks at δ = 3.5~3.8 ppm, which are two peaks of the -CH_2_-CH_2_-O-group of APEG; and peaks at δ = 1~2 ppm, which are H peaks on the main chain. From the above analysis, it can be judged that each monomer was involved in the reaction to synthesize the final product.

### 3.2. Heavy Oil Property Determination

According to the GC-MS test analysis, heavy oil contains mainly large amounts of bitumen and gum. The main components of heavy oil include Pentadecane,2,6,10,14-tetramethyl-(6.43%), Stigmastane (8.67%), .beta.-iso-Methylinone (3.8%), Coprostane (3.68%), Cholestane (13.02%), Octadecane (5.17%), Heptadecane (5.84%), Tetracosane (4.04%), etc.

### 3.3. Surface Performance

#### 3.3.1. Surface Tension

**Figure 4:**
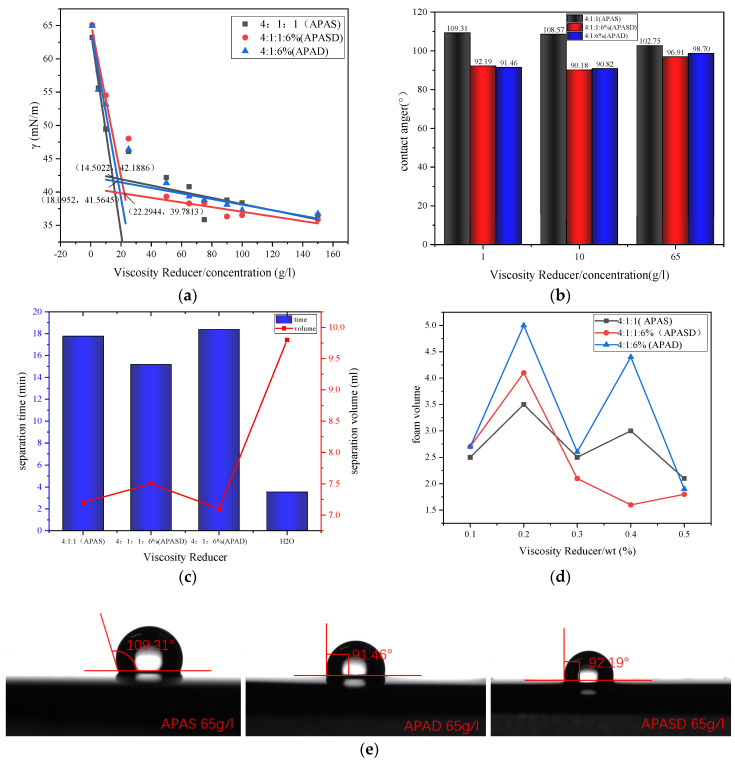
(**a**) Surface tension diagrams of three different viscosity reducers; (**b**) contact angle of three different viscosity reducers at 1 g/L, 5 g/L, and 65 g/L; (**c**) separation time and separation volume of three viscosity reducers; (**d**) foam volume of three viscosity reducers; (**e**) contact angle pictures of three viscosity-reducing agents.

The surface tension measurements of APAS, APASD, and APAD are shown in Figure 4a. The surface tensions measured for different mass concentrations of viscosity-reducing agent solutions are represented by scatter points, and two straight lines are obtained by linearly fitting the scatter points of the front and back parts, respectively. The x coordinate corresponding to the intersection point of the two lines is CMC, and the y coordinate is γ(CMC). According to the fitted results, it is known that CMC_(APAS)_ < CMC_(APAD)_ < CMC_(APASD)_ and γ(CMC)_(APASD)_ < γ(CMC)_(APAD)_ < γ(CMC)_(APAS)_. The smallest CMC_(APAS)_ is 14.5022 g/L, and the largest CMC_(APASD)_ is 22.2944 g/L. Since the hydrophobic chain lengths used in this series of viscosity reducers are the same, the heavy average molecular weights of these products are not very different, and the calculation based on the heavy average molecular weight of APAS is 55,695 [30], which can be converted to a CMC_(APAS)_ of 2.6 × 10^−4^ mol/L and a CMC_(APASD)_ of 4 × 10^−4^ mol/L, which shows that the difference between even the largest and smallest CMC is not significant. In view of γ(CMC), APASD has the best ability to reduce surface tension, while APAS has the worst ability to reduce surface tension. As we know, surfactants can reduce the surface tension of water because surfactant molecules can insert hydrophilic polar groups into the water and hydrophobic nonpolar groups into the air, displacing the liquid-gas interface by arranging them in an orderly manner at the liquid-gas interface, making the surface tension of water lower [33]. However, because ionic surfactants contain charged hydrophilic groups, resulting in repulsive forces between ionic surfactant molecules, the surfactant molecules do not align closely at the liquid-gas interface, which leads to a reduction in the ability of surfactants to reduce surface tension [34]. In the case of amphoteric surfactants or anionic and cationic surfactant complex systems, the charge of the cationic group and the charge of the anionic group can shield the electrostatic repulsion between some of the same charged groups through strong electrostatic interactions, so that the corresponding surfactant molecules can be arranged more closely than those of the anionic and cationic surfactants, i.e., the ability to reduce surface tension is better. The smaller the net charge of surfactant molecules, the stronger their ability to reduce surface tension [35,36]. For the series of polyether carboxylate viscosity reducers synthesized in this experiment, the corresponding net charge size was calculated by using the total mass and molar ratio of monomers as shown in Table 1 below, and we can see that the net charge of APAS is greater than APASD, which is greater than APAD, so APAS has the worst ability to reduce surface tension, which is consistent with the experimental results. Theoretically, APAD should have a better ability to reduce surface tension than APASD, but the experimental results are quite the opposite. The possible reason is that APASD has some of its carboxylic acid groups replaced with sulfonic acid groups compared to APAD, and since the synthesized viscosity reducer undergoes a 20% sodium hydroxide solution to adjust the pH, this leads to a large number of sodium ions in the viscosity reducer solution, and the sulfonic acid groups have better salt resistance than the carboxylic acid groups [37,38], so here APASD has a better ability to reduce surface tension than APAD.

#### 3.3.2. Contact Angle

The contact angle measurements for three mass concentration solutions of APAS, APASD, and APAD are shown in Figure 4b below. The contact angle here refers to the contact angle between the surfactant solution and the heavy oil, which can measure the wetting performance of the surfactant solution on the heavy oil. The smaller the contact angle, the better the wetting performance of the surfactant solution on the heavy oil, and the better it is for viscosity reduction [39]. According to Figure 4b, the contact angles of APAD and APASD are similar and smaller than those of APAS. This is also due to the net charge of the surfactant. When the surfactant solution droplets are in contact with the heavy oil, the hydrophobic non-polar groups of the surfactant molecules will be inserted into the heavy oil, while the hydrophilic groups are on the outside of the heavy oil, and the hydrophilic groups of the same charge will repel each other, resulting in the surfactant molecules not being arranged very closely at the water-oil interface, making the surfactant solution unable to wet the heavy oil well. The higher the net charge of the surfactant, the higher the net charge of the surfactant. The larger the net charge of the surfactant, the stronger the repulsive effect, resulting in a larger contact angle. Therefore, the contact angle of the APAS with the highest net charge is also the largest. The contact angle pictures of the three viscosity-reducing agents at a concentration of 65 g/L are shown in Figure 4e below. Figure 4b shows that the contact angles of APAD and APASD are 91.46° and 92.19°, respectively, while APAS has the largest contact angle of 109.31°.

#### 3.3.3. Water Distribution Performance

**Figure 5:**
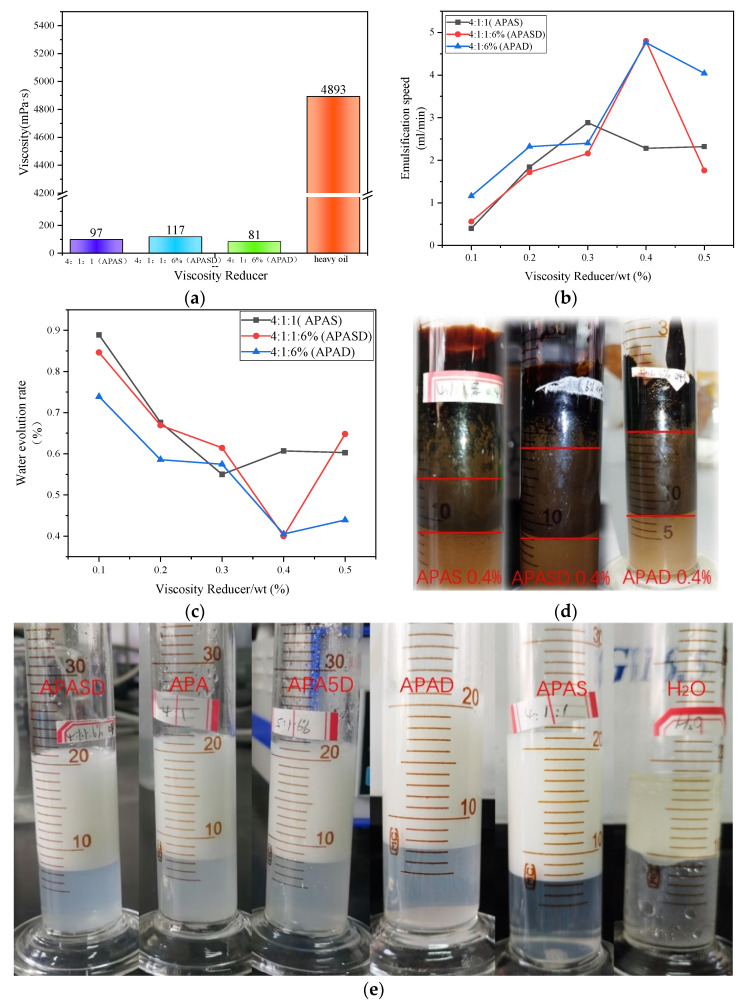
(**a**) Viscosity of three viscosity reducers after emulsification with heavy oil; (**b**) emulsification rate of three viscosity reducers; (**c**) precipitation rate of three viscosity reducers; (**d**) emulsification picture of three viscosity reducers and heavy oil; (**e**) water volume picture of five viscosity reducers.

The water separation time and water separation volume measured after emulsification of APAD, APASD, and APAD mixed with general edible oil are shown in Figure 4c below, with water as the reference group. The water separation performance reflects the stability of the emulsion formed by the emulsification of surfactant solution and oil by the volume of water dispensed; the longer the water separation time and the smaller the water separation volume, the better the stability of the emulsion and the stronger the emulsification ability. It can be seen from the graph that the time required to precipitate 5 mL of water is APAD > APAS > APASD, and the final volume of precipitated water is exactly the opposite, APAD < APAS < APASD, while the water as the reference group has the shortest partition time and the maximum final partition volume. Therefore, all three surfactant solutions have very significant emulsification ability compared to water, and the reason why APAD has better emulsification performance than APAS and APASD is still that APAD has the lowest net molecular charge, resulting in the smallest molecule-to-molecule repulsion, so more APAD molecules are densely arranged in the oil-water interface, resulting in a more stable oil-in-water emulsion [40]. The distribution volumes of the five viscosity reducers are shown in Figure 5e below.

#### 3.3.4. Foaming Performance

The maximum volume of foam generated by the three surfactants, APAS, APASD, and APAD, is shown in Figure 4d. The volume of foam produced reflects the foaming performance of the surfactants, which in turn is related to the magnitude of the surface tension of the solution; the higher the surface tension, the weaker the foaming performance [41]. As can be seen from Figure 4d, APAD produced the most foam overall at mass fractions of 0.1% to 0.5%, while APASD produced less. With mass fractions of 0.1% to 0.5%, which translates into mass concentrations of about 1 g/L to 5 g/L, the fitted straight line in the surface tension plot (Figure 4a) shows that in the range of 1 g/L to 5 g/L, APASD does have the highest surface tension and APAD has a lower surface tension, so APAD produces more foam than APAS and APASD.

### 3.4. Viscosity Reduction and Emulsification Performance

#### 3.4.1. Viscosity Reduction Performance

The viscosities of APAS, APASD, and APAD after emulsification with heavy oil are shown in Figure 5a below. It can be seen from Figure 5a that the viscosity reduction effect of the three surfactants is significant, and the viscosity reduction rate of the worst APASD is 97.61% and the best APAD is 98.34%, so the difference between them is not too big. The reason for the slight difference is the different net charges of the surfactant molecules. All three surfactants contain hydrophobic chain groups of the same chain length, and the hydrophobic non-polar groups insert into asphaltenes and gums in heavy oil to break the net structure formed by their accumulation at the oil-water interface [5,6], while the hydrophilic groups of surfactants combine with water molecules to form an aqueous layer, thus emulsifying and dispersing the heavy oil into an oil-in-water emulsion and reducing the viscosity. However, because different surfactant molecules have different net charges, the repulsion between molecules with a high net charge is also high, which means the surfactant molecules cannot be arranged closely at the oil-water interface, thus reducing the viscosity reduction performance. Therefore, the APAD with the lowest net charge has the best viscosity reduction performance. Compared with the polyoxyethylene sorbitan monooleate surfactant studied by Jana et al., APAD shows better viscosity-reducing properties [27].

#### 3.4.2. Emulsification Speed

The emulsification rates of the solutions of APAS, APASD, and APAD with different mass concentrations of the three surfactants emulsified with heavy oil are shown in Figure 5b below. It can be seen from Figure 5b that the emulsification speed of all three surfactants has a general tendency to increase with the increase in mass fraction, which precisely indicates that as the mass fraction increases, more surfactant molecules arrange on the oil-water interface, which accelerates the emulsification of heavy oil. Among them, APAD has the highest emulsification speed, which means it has the best emulsification performance. This is also due to the net charge of surfactant molecules. APAD contains the lowest net charge, so its molecule-to-molecule repulsion is the smallest, and the APAD molecules can be arranged more and more closely at the oil-water interface, which accelerates the formation of oil-in-water emulsion and improves the emulsification speed of heavy oil [42].

#### 3.4.3. Emulsion Stability

The precipitation rates of APAS, APAD, and APASD surfactant solutions formed with heavy oil are shown in Figure 5c below. It can be seen from Figure 5c that the overall water precipitation rate decreases with the increase in surfactant mass fraction, which indicates that the higher the mass fraction in the mass fraction range, the more stable the emulsion. The water precipitation rate of APAD is generally lower at all mass fractions, indicating that the emulsion formed by APAD solution is more stable. This is due to the low net charge of APAD itself. The lower net charge makes the repulsion between APAD molecules smaller, which allows more surfactant molecules to arrange at the oil-water interface, resulting in more hydrophobic non-polar groups to insert into the heavy oil and more hydrophilic groups to combine with water molecules, making the formed water layer thicker, which makes a more stable water layer between the heavy oil particles wrapped by water, so the emulsion is more stable [40]. The pictures of the emulsion formed by the three viscosity reducers with heavy oil at a mass fraction of 0.4% are shown in Figure 5d.

The above experimental analysis shows that APAD has the best viscosity reduction and emulsification performance among the three surfactants.

### 3.5. Viscosity Reduction Mechanism

Since the synthesized viscosity reducers are polyether carboxylate structures, they must contain both ether and carboxylic acid, and carboxylic acid itself is an anionic hydrophilic group. APAS containing sodium *p*-styrenesulfonate is regarded as an “anionic surfactant”, APAD containing dimethyldiallylammonium chloride is considered a “cationic surfactant”, and APASD containing both sodium *p*-styrenesulfonate and dimethyldiallylammonium chloride is regarded as an “amphoteric surfactant”. The relationship between the performance and the different electrical properties of the three surfactants, APAS, APAD, and APASD, was first discussed above. Additionally, it was concluded that the amount of the net charge of the surfactant molecule itself affects the surface performance and viscosity-reducing emulsification performance of the surfactant, and the difference between the carboxylic acid group and the sulfonic acid group also has a certain effect on the surface performance. Next, the performance of two other surfactants, APA and AP5AD, will be compared with the previous three surfactants to verify the correctness of the conclusion.

#### 3.5.1. Surface Tension

**Figure 6:**
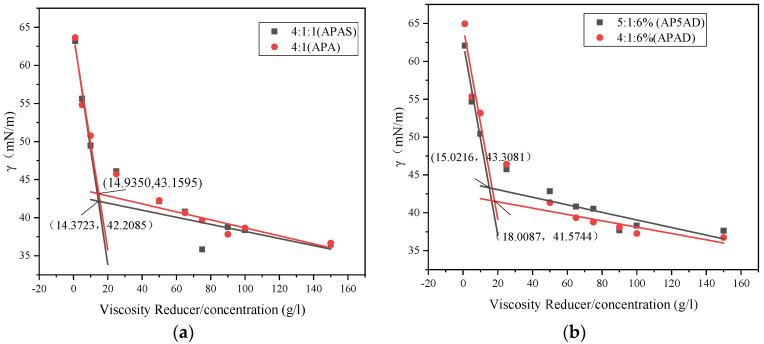

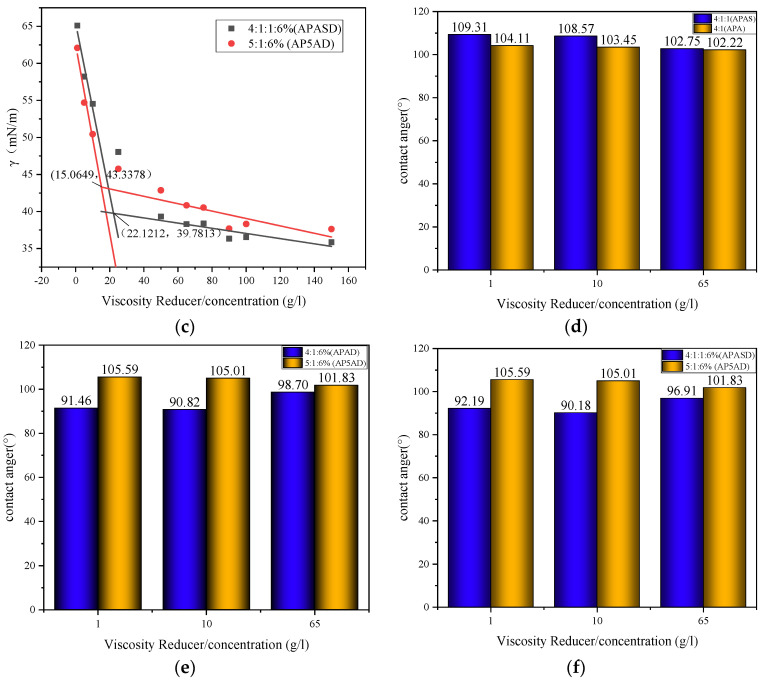
(**a**) Surface tension between APAS and APA; (**b**) surface tension between AP5AD and APAD; (**c**) surface tension between APASD and AP5AD; (**d**) contact angle between APAS and APA; (**e**) contact angle between APAD and AP5AD; (**f**) contact angle between APASD and AP5AD.

The surface tension scatter plots of APAS and APA are shown in Figure 6a below. The graph shows that the difference between the two CMCs is very slight, and γCMC_(APAS)_ < γCMC_(APA)_. APA has no sulfonic acid group compared to APAS, and according to the above table, the net charge of the APA molecule is lower than APAS, and the surface tension of APA at CMC should be lower than APAS in theory, but the result is just the opposite, indicating that the sulfonic acid group plays a role. In a solution containing a large number of sodium ions, the sulfonic acid group is better than the carboxylic acid group due to its salt resistance, so APAS containing the sulfonic acid group has a stronger ability to reduce surface tension than APA.

The surface tension scatter plots of AP5AD and APAD are shown in Figure 6b below. The difference in CMC between the two can be seen in Figure 6b, with γCMC_(APAD)_ < γCMC_(AP5AD)_. AP5AD has one more share of the carboxylic acid group compared to APAD, which also leads to a higher net charge of AP5AD than APAD, so AP5AD is not as strong as APAD in reducing surface tension.

The surface tension scatter plots of APASD and AP5AD are shown in Figure 6c below. Figure 6c also shows that γCMC_(APASD)_ < γCMC_(AP5AD)_, and AP5AD not only has no sulfonic acid group compared to APASD but also adds a carboxylic acid group. This leads to the net charge of AP5AD being larger than APASD and the excellent salt resistance performance of APASD’s sulfonic acid group, which makes the surface tension difference between the APASD solution and the AP5AD solution larger than the other groups. It speculates that the net charge of surfactant molecules and the role of sulfonic acid groups are superimposed on each other, resulting in a wider surface tension difference.

#### 3.5.2. Contact Angle

The contact angles of APAS and APA are shown in Figure 6d below. It indicates that the contact angles of both APAS are larger than APA because the net charge of APAS molecules is higher than APA, resulting in greater repulsion between APAS molecules and APAS molecules, so their contact angles are also larger, which is consistent with the conclusion.

The contact angles of APAD and AP5AD are shown in Figure 6e below. It indicates that the contact angle of AP5AD is higher than that of APAD, which is also caused by the fact that the net charge of the APAD molecule itself is smaller than that of AP5AD, which is also consistent with the conclusion.

The contact angles of APASD and AP5AD are shown in Figure 6f below. It shows that the contact angle of APASD is lower than that of AP5AD because the net charge of APASD is smaller than that of AP5AD, which is consistent with the conclusion.

#### 3.5.3. Water Separation Performance

**Figure 7:**
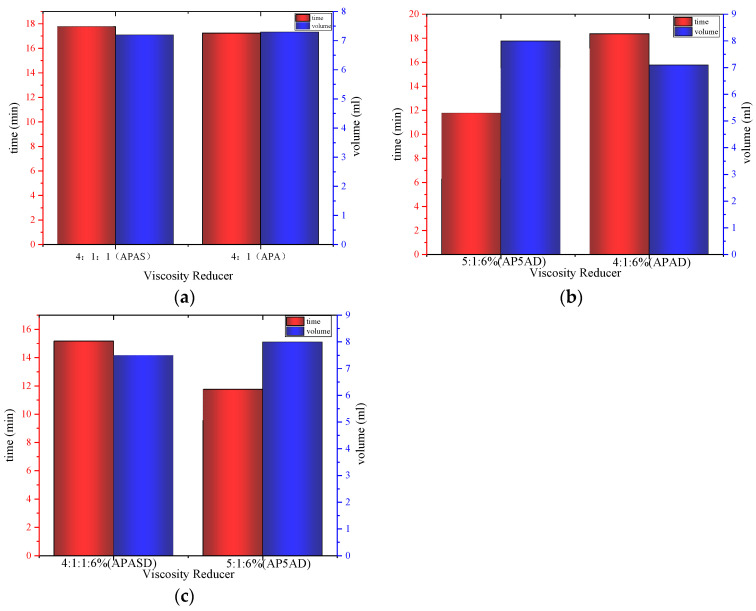
(**a**) Partition time and partition volume of APAS and APA; (**b**) partition time and partition volume of AP5AD and APAD; (**c**) partition time and partition volume of APASD and AP5AD.

The partition time and partition volume of APAS and APA, AP5AD and APAD, and APASD and AP5AD are shown in Figure 7a–c below, respectively. It shows that the partition time of APAS is longer than APA and the partition volume is smaller than APA, indicating that the emulsion formed by the APAS surfactant and edible oil is more stable. Although the net charge of APAS is higher than that of APA, the presence of the sulfonic acid group makes the stability of the emulsion formed by APAS a little better than that of APA.

In contrast, the partition time of APAD was longer and the partition volume was smaller than that of AP5AD, indicating that the emulsion formed by the APAD surfactant with edible oil was more stable. The partition time of APASD was longer and the partition volume was smaller than that of AP5AD, indicating that the emulsion formed by APASD with edible oil was more stable than that of AP5AD. This is also because the net charge of APASD and APAD is smaller than that of AP5AD, which is consistent with the conclusion.

After the comparison of surface tension, contact angle, and water separation performance tests, it was found that the experimental phenomena were consistent with the mechanism assumed above, further illustrating the correctness of the conclusions.

## 4. Conclusions

In this paper, we synthesized different electrical polyether carboxylate viscosity reducers, and through a series of tests and comprehensive analysis, we concluded that APAD has the best viscosity reduction performance and can reduce the viscosity of heavy oil to 81 mPa·s. APAD also had the best emulsification performance, with the greatest emulsification speed and the best emulsion stability.

The performance test of each polyether carboxylate viscosity reducer reasonably explains how the difference in electrical properties affects the viscosity reduction performance of surfactants. That is, the amount of the net charge of the surfactant itself affects the repulsion between the surfactant molecules, and the higher the net charge, the greater the repulsion, which means the surfactant molecules cannot closely arrange themselves between the oil-water interface or the liquid-gas interface, resulting in a reduction in the emulsification viscosity reduction performance and surface properties.

The shortcoming is that there is no deeper understanding of whether the net charge size of the surfactant itself or the difference between the sulfonic and carboxylic acid groups plays a greater role in reducing surface tension, which will be the focus of future work.

## Figures and Tables

**Table 1:** Net charge of five different viscosity-reducing agent molecules.

Surfactant	APAS	APASD	APAD	APA	AP5AD
Net charge (mol)	0.13705	0.134225	0.131635	0.1352	0.152646

## Data Availability

Not applicable.
